# Comprehensive GC/MS Profiling of Volatile Organic Compounds in Whole and Glandular Saliva Using MonoTrap Micro-Extraction

**DOI:** 10.3390/metabo15110726

**Published:** 2025-11-06

**Authors:** Asuka Mori, Masae Kuboniwa, Eiichiro Fukusaki

**Affiliations:** 1 Department of Biotechnology, Graduate School of Engineering, The University of Osaka, Osaka 565-0871, Japan; asuka_mori@bio.eng.osaka-u.ac.jp; 2 Department of Preventive Dentistry, Graduate School of Dentistry, The University of Osaka, Osaka 565-0871, Japan; kuboniwa.masae.dent@osaka-u.ac.jp; 3 Osaka University Shimadzu Omics Innovation Research Laboratories, The University of Osaka, Osaka 565-0871, Japan

**Keywords:** saliva, glandular saliva, volatile compound, VOC, GC/MS, MonoTrap, oral microbiome

## Abstract

Background/Objectives: Salivary volatile organic compounds (VOCs) are promising noninvasive biomarkers for a wide range of diseases. While glandular saliva, secreted by salivary glands, is a relatively pure biofluid, whole saliva is a complex mixture containing oral microbiota, food debris, and desquamated epithelial cells. Therefore, a comprehensive comparison of the VOC profiles of these two types of saliva is essential to identify biologically relevant compounds. In this study, we aimed to establish a reliable method for VOC profiling from small saliva volumes and identify VOCs that reflect the biological differences between glandular and whole saliva. Methods: We developed a protocol combining MonoTrap extraction with dichloromethane, allowing the analysis of VOCs from just 100 µL of saliva. To address the issue of sampling-derived artifacts, we implemented a two-step blank analysis to systematically exclude compounds originating from the collection device. Results: Our analysis successfully identified a total of 72 VOCs. Following blank analysis, we systematically excluded 15 artifacts originating from the sampling device. Subsequent orthogonal partial least squares discriminant analysis (OPLS-DA) and Wilcoxon signed-rank test (using variable importance for prediction (VIP) > 1.0 and *q* < 0.05) identified 10 key VOCs that were significantly higher in whole saliva than in glandular saliva. These compounds included isobutyric acid, isovaleric acid, 4-methylvaleric acid, 3-phenylpropionic acid, indole, skatole, methyl mercaptan, 1-propanol, δ-valerolactam, and acetaldehyde. Most of these compounds originate from the metabolic activities of the oral microbiome, suggesting that the distinct VOC profile of whole saliva is predominantly influenced by microbial activity. Conclusions: Our findings demonstrated the effectiveness of this method for identifying biologically relevant VOCs from relatively small sample volumes. The identified VOC profiles highlight the contribution to the discovery of non-invasive biomarkers for oral health and serve as a solid foundation for future research into clinical applications.

## 1. Introduction

Volatile organic compounds (VOCs) are diverse low-molecular-weight organic compounds produced during metabolic activities within the body. They are present in trace amounts in various biological samples, including breath, saliva, sweat, and blood [1]. VOC profiles in these clinical samples show promise as non-invasive biomarkers reflecting an individual’s health status and the presence of disease-specific metabolic abnormalities [2,3,4]. Among these, saliva is an ideal specimen as it can be collected without invasive procedures such as blood sampling, and the collection does not place a burden on the patient. However, VOCs in saliva are generally present at very low concentrations [5], and high-sensitivity analyses currently require large sample volumes. This not only compromises the advantages of non-invasive sampling but also hinders the analyses, particularly in cases where obtaining large sample volumes is difficult, such as with children, older adults, or patients with specific diseases. Therefore, establishing pretreatment methods capable of efficiently analyzing VOCs in small samples is an urgent priority for VOC research. Conventional VOC extraction methods, such as the headspace method and solid-phase microextraction have difficulties in comprehensively profiling trace VOCs in small aqueous samples due to the influence of moisture and limitations in extraction efficiency [6]. In contrast, MonoTrap, with its porous monolithic structure, has a large adsorption capacity due to its large surface area [7]. Furthermore, the use of dichloromethane is expected to enable its dispersion within the hydrophobic structure of the MonoTrap. This suggests the potential for this approach to serve as a highly efficient micro liquid–liquid extraction system for trace aqueous samples, thereby enabling comprehensive VOC profiling.

Furthermore, the sampling method complicates the interpretation of the VOC analysis. Commercially available devices, such as the Salivette, are widely used for saliva collection [8]. However, there is a potential risk of contamination from VOCs originating from the device itself, as well as adsorption and loss of specific compounds [9]. Consequently, unless device-derived artifacts are properly excluded, interpreting the resulting VOC profile as being of “biological origin” is difficult. To enhance the reliability of salivary VOC research, these sampling-related issues must be addressed. 

The complex composition of human saliva makes VOC analyses challenging. Human saliva is a complex matrix containing not only fluid from the salivary glands, but also gingival crevicular fluid, oral bacteria, desquamated epithelial cells, food debris, and other components [10,11]. Therefore, VOCs in whole saliva are considered a mixture of those derived from human metabolism, oral microorganisms, and exogenous factors (such as diet). Identifying the VOCs derived from the metabolic activity of oral bacteria within this complex matrix and distinguishing them from human-derived or exogenous compounds is extremely difficult. Contrastingly, glandular saliva, directly collected from salivary glands, is a relatively pure biofluid with minimal contamination from the oral environment by microorganisms and food debris [10]. Therefore, to identify VOC biomarkers that reflect the metabolism of oral bacteria, it is essential to compare the VOC profile of glandular saliva, which has minimal bacterial contamination, with that of whole saliva, a complex matrix containing various components of the oral environment, collected from the same individuals. Studies have already been conducted on non-volatile metabolites [10,11] and proteins [12] to determine the differences between whole and glandular saliva. In contrast, prior research on salivary VOCs has been strictly limited to whole saliva [13,14,15]. Consequently, the differential VOC profiles originating from the relatively pure glandular saliva versus the whole saliva, which reflects the overall oral environment, remain an unexplored knowledge gap. We hypothesized that VOCs enriched in whole saliva originate primarily from oral microbial metabolism.

To address these challenges, we set two primary objectives in this study. First, we aimed to develop a new sample preparation method to efficiently extract VOCs from small saliva samples. Our method, which combines MonoTrap extraction with dichloromethane, enabled comprehensive VOC profiling from just 100 µL of saliva. Second, using this method, we aimed to perform a detailed comparison of the VOC profiles of whole and glandular saliva to identify biomarkers that reflect the metabolic activities of the oral microbiome. By further incorporating a two-step blank analysis using both saliva and ultrapure water as samples, we systematically excluded device-derived artifacts and achieved a more reliable exploration of biomarkers. This study not only provides crucial insights for establishing salivary VOCs as reliable biomarkers but also demonstrates a novel approach for VOC analysis from micro-volume samples; this can aid other research using clinical specimens, such as tears and sweat. 

## 2. Materials and Methods

### 2.1. Participants

This study was approved by the Human Ethics Committee of the Osaka University Dental Hospital (approval no. R4-E10-2) and was conducted in accordance with the Declaration of Helsinki. Written informed consent was obtained from all the participants. Ten Japanese participants were recruited for this study. The participant ID, age, and sex are presented in Table 1. The main objective of this study was to compare the VOC profiles of glandular saliva and whole saliva samples collected from nine participants, except participant 17. A preliminary examination was conducted using whole saliva samples collected from participant 17 to evaluate the impact of Salivette (Sarstedt, Nümbrecht, Germany) on VOC profiles.

### 2.2. Sample Collection and Preparation

#### 2.2.1. Main Experiment

All saliva samples were collected between 11:00 a.m. and 12:00 p.m. in the following order: glandular saliva followed by whole saliva. The participants were instructed to refrain from eating or drinking anything other than water for 8 h prior to saliva collection. Then, they were instructed to refrain from eating, drinking (including water), and brushing their teeth for 3 h prior to saliva collection. Glandular and whole saliva were collected using the procedure described below.

For glandular saliva collection, the participants first rinsed their mouths with water. Next, with the mouth open and tongue pressed against the upper palate, a Salivette cotton swab was placed at the outlet of the submandibular/sublingual gland using disposable tweezers (AS ONE, Osaka, Japan) (Figure S1). The swab was kept in place for 5 min with the mouth closed (For participant 01, owing to insufficient saliva volume, the swab was kept in place for 15 min). Subsequently, the cotton swab was removed using disposable tweezers and placed in a Salivette tube. Glandular saliva was collected by centrifugation of the Salivette tube at 4 °C and 1000× *g* for 2 min. The obtained glandular saliva was aliquoted into 2.0 mL microtubes (Eppendorf, Hamburg, Germany) in 200 µL aliquots. Each aliquot was used only once to prevent freeze–thaw cycles. The samples were frozen in liquid nitrogen and stored at −80 °C for a maximum of 1.5 months until analysis.

For whole saliva collection, unstimulated whole saliva was expectorated over a 5 min period into a 50 mL tube (Corning, New York, NY, USA) kept on ice. After 15 min on ice, the aqueous layer was collected. The obtained whole saliva was aliquoted into 2.0 mL microtubes by 200 µL, frozen in liquid nitrogen, and stored at −80 °C until analysis following the detailed protocol described above.

#### 2.2.2. Preliminary Experiment for Evaluation of the Effects of Salivette

To investigate the effect of Salivette use on saliva VOC profiles, we compared the VOC profiles of whole saliva with and without Salivette treatment, collected from a single participant (Participant 17). To further exclude compounds originating from the Salivette, a complementary analysis was performed by comparing the VOC profiles of ultrapure water with and without Salivette treatment.

Whole saliva was collected without Salivette treatment, following the same procedure as in the main experiment. Unstimulated whole saliva was expectorated over a 5 min period into a 50 mL tube kept on ice. After 15 min on ice, the aqueous layer was collected. The collected whole saliva was aliquoted into 2.0 mL microtubes by 200 µL, frozen in liquid nitrogen, and stored at −80 °C until analysis. 

For whole saliva collection with Salivette treatment, a Salivette cotton swab was placed inside a 50 mL tube prior to use. Saliva was collected in tubes for 5 min. Subsequently, the cotton swab was removed using disposable tweezers and placed in a Salivette tube. Whole saliva was collected by centrifugation of the Salivette tube at 4 °C and 1000 *g* for 2 min. The obtained saliva was aliquoted into 2.0 mL microtubes at 200 µL each, frozen in liquid nitrogen, and stored at −80 °C until analysis.

### 2.3. VOC Extraction and Gas Chromatography/Mass Spectrometry (GC/MS) Analysis

#### 2.3.1. VOC Extraction

The following preparations were made prior to the extraction of VOCs from saliva. Unless otherwise stated, all procedures were conducted at a room temperature of 22–24 °C. First, the MonoTrap RGPS TD (GL Sciences, Tokyo, Japan) was cut in half. This modification was essential because the total length of the MonoTrap (10 mm) exceeded the liquid level (approximately 6 mm) of the 100 µL saliva sample when placed in the glass insert. Cutting the MonoTrap in half (to approximately 5 mm) ensured its complete immersion in the micro-volume sample, thereby maximizing the contact surface area and optimizing the adsorption kinetics. The cut MonoTrap was conditioned with nitrogen gas flowing at approximately 45 mL/min for 2 h at 270 °C before use. All saliva samples were thawed at room temperature before use. 

All saliva samples were subjected to the following VOC extraction protocols. The main experiment included 18 samples (glandular [*n* = 9] and whole saliva [*n* = 9]). The preliminary experiment for evaluating the effects of Salivette included six samples (whole saliva collected with and without Salivette; *n* = 3, technical replicates). 

A 0.2 mL flat-bottom glass insert (GL Sciences) was placed in a 2 mL clear vial (Thermo Fisher Scientific, Waltham, MA, USA). MonoTrap was then inserted into the glass insert, and 50 µL of dichloromethane was added. After vortexing for 10 s, the vials were kept at room temperature for 20 min. Subsequently, the MonoTrap was transferred to a new 2 mL clear vial containing a new glass insert with 100 µL of saliva sample. After vortexing for 10 s, the vial was maintained at room temperature for 60 min to extract VOCs from the saliva. During this process, vortex mixing (10 s) was performed every 20 min. The MonoTrap was transferred to a liner (GL Sciences) and subjected to thermal desorption-GC/MS analysis.

#### 2.3.2. GC/MS Analysis

GC/MS analysis was performed using a GCMS-TQ8050 NX (Shimadzu, Kyoto, Japan) equipped with a multifunctional autosampler AOC-6000 (Shimadzu) and a multimode GC inlet OPTIC-4 (GL Sciences). OPTIC-4 inlet temperature program was as follows: It was first increased from an initial temperature of 40 °C to 50 °C at a rate of 5 °C/s, held at 50 °C for 240 s, then increased to 250 °C at 5 °C/s, and held at 250 °C for 360 s. The cryotrap was used at −120 °C for 600 s and then rapidly increased to 250 °C at 30 °C/s. The column flow was set to 1.0 mL/min for 240 s and then increased to 2.3 mL/min until the end of the analysis. The split flow was set to 230 mL/min for 240 s and then changed to 2.3 mL/min until the end of the analysis. The septum purge flow was set to 5 mL/min. The inlet temperature was maintained at 50 °C, and the split flow was set to a high rate of 230 mL/min for the initial 240 s to facilitate venting of the residual dichloromethane. GC analysis was performed using InertCap FFAP (60 m × 0.32 mm i.d., df = 0.50 µm; GL Sciences) with helium as the carrier gas. The column oven temperature conditions were as follows: it was maintained at 40 °C for 3 min, raised to 250 °C at 3 °C/min, and finally kept at 250 °C for 30 min. The MS was operated in the scan mode (*m*/*z* 24–350) using electron ionization (ionization energy: 70 eV) with an event time of 0.200 s. The ion source temperature and interface temperature were set to 250 °C. To increase the sensitivity, the detector voltage was raised by 0.2 kV in relative value. A standard mixture of alkanes (C7–C26) was used to calculate the retention index (RI). 

### 2.4. Data Analysis

The raw data obtained from the analysis were converted to netCDF files using GCMSsolution ver. 4.20 (Shimadzu), and then to ABF files using an ABF converter (Reifycs, Tokyo, Japan). MS-DIAL ver. 4.9.221218 (RIKEN, Saitama, Japan) was used for baseline correction, noise removal, peak detection, alignment, and automatic annotation based on the RI and mass spectral information of the compounds. Compound identification was based on the similarities of experimental RI and mass spectrum to those of compounds registered in the in-house library. Our in-house library comprises 572 compounds. A list of the compounds with their names and CAS registry numbers is summarized in Table S1. The MS-DIAL analysis conditions were set as follows: minimum peak height: 5000 amplitude, smoothing method: linear weighted moving average, EI similarity library tolerance: 70, and RI tolerance: 20.

To compare the VOC profiles between glandular and whole saliva samples, orthogonal partial least squares discriminant analysis (OPLS-DA) was performed using MetaboAnalyst 6.0, and the Wilcoxon signed-rank test was conducted using R (ver. 4.5.1). To identify Salivette-derived compounds, the peak intensities were compared between samples with and without Salivette treatment using either Student’s *t*-test or Welch’s *t*-test, depending on data variance (Statistical analysis tool: https://systemsomicslab.github.io/compms/others/main.html#Statistics, accessed on 16 September 2025). This comparison was performed on whole saliva samples from the preliminary experiment and blank ultrapure water samples. All plots (bars, boxes, and paired scatter plots) were generated using R.

## 3. Results

### 3.1. Evaluation of the Effects of Salivette on Whole Saliva VOC Profiles

**Figure 1:**
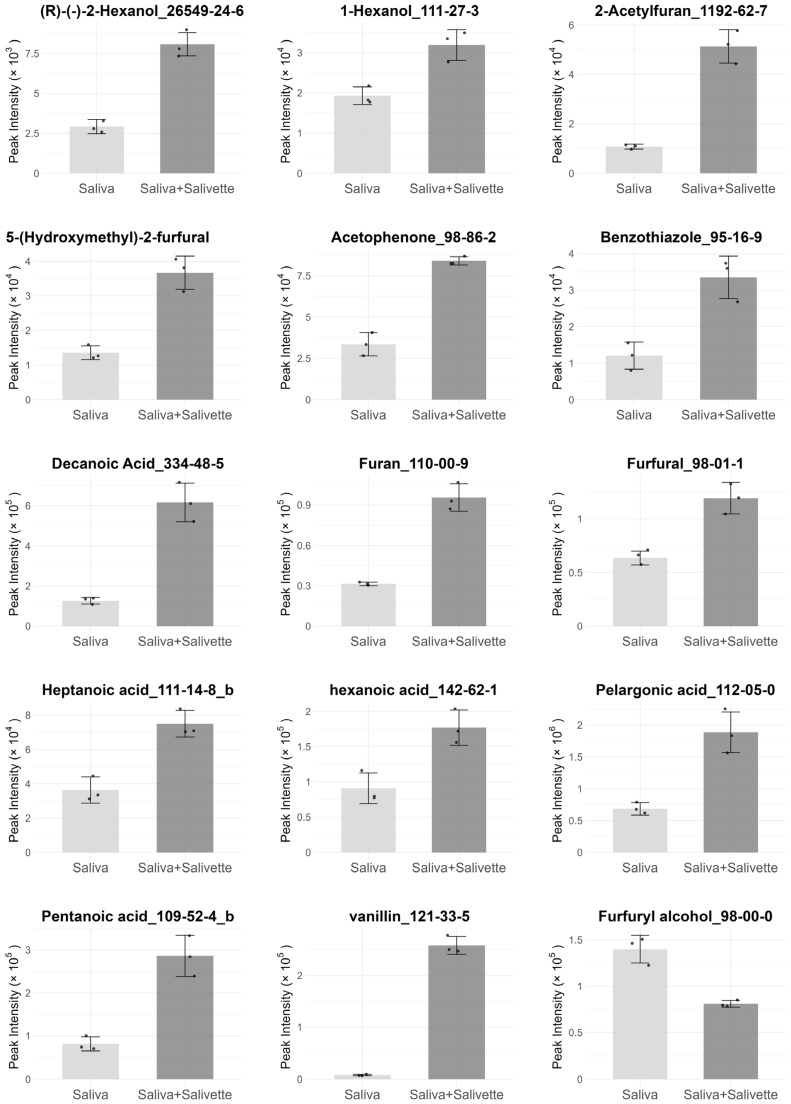
Bar plot of 15 compounds significantly changed upon using Salivette (Saliva: whole saliva collected without Salivette, Saliva+Salivette: whole saliva collected with Salivette).

First, to investigate the effects of Salivette on the VOC profiles of saliva, we compared the VOC profiles of whole saliva collected from the same participants with and without Salivette treatment. A total of 62 compounds, consisting of alcohols (13), aromatic compounds (17), aldehydes (6), fatty acids (13), ketones (7), volatile sulfur compounds (VSCs) (3), and others (3), were detected in six samples: whole saliva collected with Salivette (*n* = 3), and whole saliva collected without Salivette (*n* = 3) (Table S2). Among these, compounds showing changes in peak intensity, with or without Salivette treatment, were investigated. The results indicated that 14 compounds (2-hexanol, 1-hexanol, 2-acetylfuran, 5-hydroxymethyl-2-furaldehyde, acetophenone, benzothiazole, decanoic acid, furan, furfural, heptanoic acid, hexanoic acid, pelargonic acid, valeric acid, and vanillin) significantly increased with Salivette use, whereas furfuryl alcohol showed a significant decrease (*t*-test, *p* < 0.05, *n* = 3) (Figure 1). The 14 compounds are possible blank compounds originating from the Salivette, whereas the furfuryl alcohol is a compound potentially adsorbed to the Salivette. Therefore, these 15 compounds were excluded from subsequent analysis comparing the VOC profiles of whole and glandular saliva.

### 3.2. Comparison of VOC Profiles in Whole and Glandular Saliva

Next, we compared the VOC profiles of glandular and whole saliva. Both saliva samples were collected from the nine participants and subjected to GC/MS analysis, where a total of 72 compounds were detected. Of these, 15 compounds that showed significant changes owing to Salivette use were excluded from subsequent analysis. The remaining 57 compounds included alcohols (11), aromatic compounds (15), aldehydes (6), fatty acids (10), ketones (6), VSCs (4), and others (5) (Table S3). 

**Figure 2:**
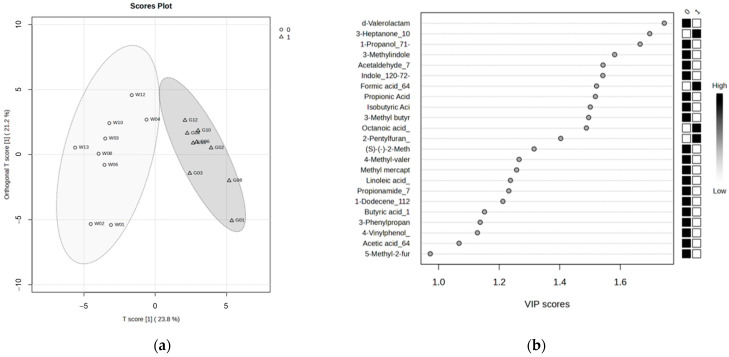
(**a**) Score plot for OPLS-DA (W: whole saliva, G: glandular saliva); (**b**) VIP scores for OPLS-DA.

**Figure 3:**
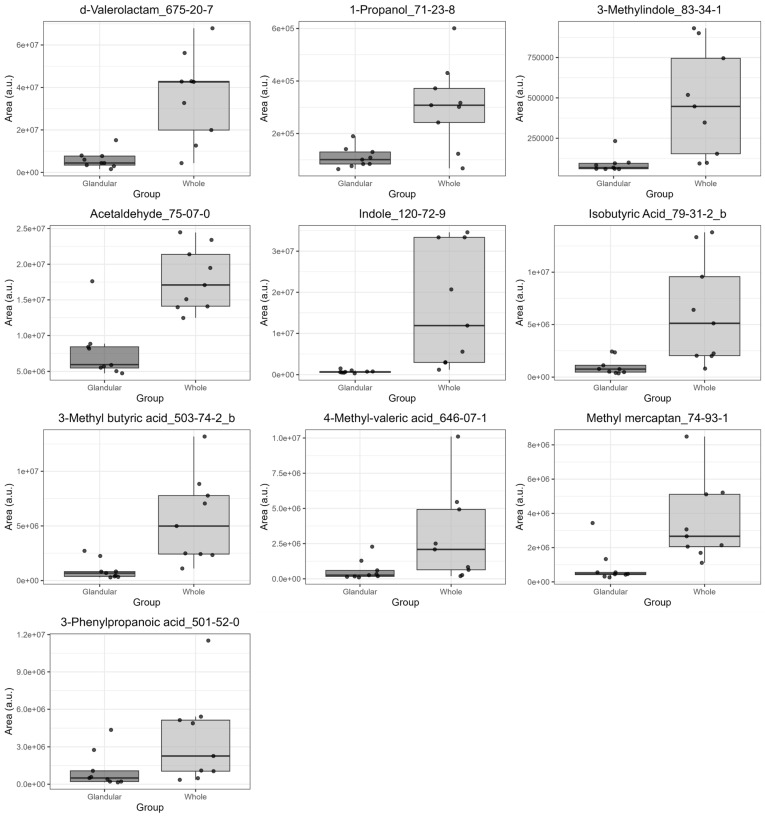
Box plots of 10 compounds showing significant changes (*q* < 0.05, Wilcoxon signed-rank test) between glandular and whole saliva.

To identify differences in VOC profiles between glandular and whole saliva, OPLS-DA was performed using these 57 components as explanatory variables (Figure 2). The R^2^Y and Q^2^ were 0.743 and 0.679, respectively, indicating high model performance. Consequently, 22 compounds with variable importance for prediction (VIP) > 1.0, which is the conventional threshold for screening components contributing to group separation, were identified. Since VIP > 1.0 alone is considered a preliminary screening criterion, we further evaluated the validity of these top VIP compounds. Wilcoxon signed-rank tests were performed to confirm whether the peak intensity of each compound showed a statistically significant difference between the two saliva samples. The results indicated significant differences (*q* < 0.05) for most of the compounds with high VIP values (17 of 22 compounds). Next, to exclude the possibility that these high-VIP compounds were blank compounds, we performed a blank analysis of ultrapure water treated with Salivette for comparison. These results suggested that seven compounds, including 3-heptanone, were likely Salivette-derived blank compounds. Additionally, some compounds, including formic acid, were not detected in the blank samples; thus, it could not be determined whether they originated from the Salivette. These verification results are summarized in Table 2, including the VIP values, false discovery rate from the Wilcoxon signed-rank test, and Salivette blank evaluation for each compound. Based on these verifications, we concluded that 10 compounds with statistically significant differences that did not originate from sampling reflected the biological differences between glandular and whole saliva. The 10 compounds were δ-valerolactam, 1-propanol, skatole, acetaldehyde, indole, isobutyric acid, isovaleric acid, 4-methylvaleric acid, methyl mercaptan, and 3-phenylpropionic acid. The box and paired scatter plots of these compounds are shown in Figure 3 and Figure S2, respectively.

## 4. Discussion

In this study, we established a sample preparation method combining MonoTrap extraction with dichloromethane, enabling the profiling of VOCs from relatively small amounts of saliva sample (100 µL). This method offers high efficiency in terms of micro-volume capability and throughput, requiring only one hour for the extraction step. In this protocol, dichloromethane served a dual purpose: as an extraction solvent and a means to immerse the hydrophobic, porous polydimethylsiloxane/graphite-based MonoTrap into an aqueous saliva sample [16,17]. We hypothesize that the presence of dichloromethane significantly enhances adsorption kinetics by exploiting the unique structure of MonoTrap. Specifically, dichloromethane disperses within the MonoTrap’s massive porous structure, which dramatically increases the effective interfacial area between the aqueous sample and the VOC adsorption medium, thereby facilitating rapid VOC mass transfer and extraction. This synergistic dual functionality proved to be highly effective, enabling the reliable and comprehensive VOC profiling from small saliva samples. 

Using this VOC extraction protocol, we addressed our primary objective of identifying VOCs that reflected the biological differences between glandular and whole saliva. In this study, glandular saliva was collected using a Salivette, and whole saliva was collected by spitting into tubes. Ideally, a sampling method that minimizes the introduction of artifacts from collection devices is preferable. However, as the use of Salivette is essential for the effective collection of glandular saliva, it is necessary to properly evaluate the artifacts derived from this sampling device. As suggested by Bosman et al., Salivette use may affect the analytical results for some metabolites in the saliva [9]. While their work focused on non-volatile compounds, the issue of sampling-derived artifacts was equally relevant to VOC analysis. To address this issue, we implemented a two-step blank evaluation process. First, we compared whole saliva samples with and without Salivette treatment to exclude compounds whose peak intensities changed with Salivette use. Second, we excluded the compounds that changed in the case of Salivette treatment of ultrapure water from those that contributed to the difference between glandular and whole saliva. 

In this blank evaluation, most of the compounds identified as blanks increased with Salivette use. Among them, furfural and 5-hydroxymethyl-2-furaldehyde are widely recognized as degradation products of cellulose [18,19]. Given that cotton is a plant-derived material consisting mainly of cellulose, the possibility that these compounds originated from Salivette’s cotton swabs cannot be excluded. Similarly, the simultaneous increase in short- and medium-chain fatty acids (C5–C10) and alcohols (1-hexanol and 2-hexanol) is consistent with leachables from the polyethylene closure of the Salivette. A previous study demonstrated that these compounds could be detected in polyethylene packaging materials [20]. The presence of acetophenone is also supported by a previous study that reported its detection in polyethylene and polypropylene materials used in food packaging [21]. Taken together, these results suggest that the compounds associated with Salivette use are derived from either the cotton swab or the plastic container. In contrast to other compounds, furfuryl alcohol showed a decrease with the use of Salivette, suggesting that it adsorbs onto cotton swabs or plastic containers. 

Our blank analysis successfully identified 10 VOCs that were not derived from the sampling method. All these compounds showed significantly higher levels in whole saliva than in glandular saliva. These differences are likely attributable to the unique biological environment of the oral cavity, including the presence of oral microbiota, food debris, and desquamated epithelial cells [22]. 

For instance, indole and skatole are produced by tryptophan metabolism in oral bacteria such as the Gram-negative anaerobic bacteria *Prevotella intermedia*, *Porphyromonas gingivalis*, and *Fusobacterium nucleatum* [23,24]. Our previous study identified indole in the culture supernatants of *F. nucleatum* and *P. gingivalis* with strong odor intensity by GC/olfactometry, suggesting a potential role in halitosis [17]. Similarly, short-chain fatty acids are produced by the breakdown of proteins and their subsequent deamination by anaerobic bacteria in the oral cavity [25]. Isobutyric and isovaleric acids, which were significantly increased in whole saliva in this study, have been detected in bacterial isolates of *P. gingivalis*, *P. asaccharolytica*, *P. intermedia*, and *F. nucleatum* [25]. 4-Methylvaleric acid, also a branched-chain fatty acid, was significantly increased in whole saliva. However, the specific metabolic pathway of this compound in the oral cavity has not been well documented. On the other hand, methyl mercaptan is known to be a major causative compound of halitosis, given its low odor threshold (5.1 × 10^−13^ ppm) [24,26]. It is produced by anaerobic bacteria such as *Bacteroides* spp., *Eubacterium* spp., *Fusobacterium* spp., *Porphyromonas* spp., and *Treponema denticola* [24]. 

The presence of δ-valerolactam in whole saliva was also noteworthy. Although its metabolic pathway in the oral cavity is not yet fully elucidated, it has been speculated that δ-valerolactam could be a product of cadaverine metabolism, a known lysine degradation product in saliva [27]. A previous study reported the detection of δ-valerolactam in whole saliva collected using the passive drool method [28]. Given these findings, the significant increase of δ-valerolactam in whole saliva likely reflects its production via the metabolic activities of oral bacteria. In this study, 3-phenylpropionic acid was also significantly increased in whole saliva. A previous study reported that levels of this compound are significantly higher in patients with periodontitis than in healthy participants [29]. Furthermore, a significant correlation between 3-phenylpropionic acid and oral microorganisms was found [29]. Taken together, these findings strongly suggest that the significant increase of 3-phenylpropionic acid in whole saliva reflects its production via the metabolic activities of the oral microbiome. Similarly, the presence of 1-propanol can be attributed to oral microbial activity. It is believed that 1-propanol is produced at higher concentrations by microorganisms in the mouth rather than endogenously [30]. It has been suggested that this is a result of bacterial fermentation of threonine, a process that does not occur in glandular saliva [31]. Finally, we discuss the presence of acetaldehyde in whole saliva. The origin of this compound is complex and is likely influenced by a combination of multiple factors. Although acetaldehyde can be produced from ethanol in epithelia, much higher levels are derived from the metabolism of ethanol by oral bacteria [32]. Smoking and drinking also affect its production [32]. Therefore, the elevated levels of acetaldehyde in whole saliva in our study likely reflected the combined effects of microbial activity, host metabolism, and exogenous factors. The novelty of the present study lies in the application of glandular saliva as a control to eliminate systemic host-derived factors. Based on this rigorous methodology, we have robustly demonstrated the presence of microbially derived VOCs whose origins had previously been speculative, thereby providing clear differentiation from existing knowledge. Taken together, the findings on these 10 compounds suggest that the distinct VOC profile of whole saliva is predominantly influenced by microbial metabolism in the oral cavity. 

Despite these significant findings, this study has some limitations. The relatively small sample size, which was sufficient to demonstrate clear differences between glandular and whole saliva, suggests that further validation using a larger cohort is required to generalize these findings. Furthermore, our two-step blank analysis successfully excluded major sampling-derived artifacts, leading to the identification of 10 VOCs that likely originated from oral bacteria. However, we cannot entirely exclude the possibility of other unidentified artifacts.

## 5. Conclusions

This study successfully establishes a validated micro-volume MonoTrap–GC/MS protocol for reliable VOC profiling from relatively small saliva samples, which can be extended to other low-volume biological fluids. Through a robust two-step blank analysis, we successfully excluded artifacts derived from the sampling device. This approach enabled us to identify a unique and biologically relevant VOC profile characteristic of whole saliva, which was distinct from that of glandular saliva. These VOC differences between glandular and whole saliva underscore microbial contributions; however, larger microbiome-integrated studies are required for clinical validation.

## Figures and Tables

**Table 1:** Participants’ information (ID, age, and sex).

ID	Age	Sex
01	72	M
02	44	F
03	48	F
04	30	M
06	49	F
08	22	M
10	22	M
12	24	F
13	23	F
17	27	F

**Table 2:** List of compounds with VIP > 1.0 (VIP scores, results of the Wilcoxon signed-rank test, and Salivette blank evaluation).

Compound	OPLS-DA VIP	Wilcoxon Signed-Rank Test (*q* < 0.05)	Salivette Blank Evaluation *
δ-Valerolactam	1.75	0.028	No
3-Heptanone	1.70	0.016	Yes
1-Propanol	1.67	0.016	No
Skatole	1.58	0.016	No
Acetaldehyde	1.54	0.016	No
Indole	1.54	0.016	No
Formic acid	1.52	0.016	**
Propionic acid	1.52	0.035	Yes
Isobutyric acid	1.50	0.016	No
Isovaleric acid	1.50	0.016	No
Octanoic acid	1.49	0.016	Yes
2-Pentylfuran	1.40	0.016	**
2-Methyl-1-butanol	1.32	0.086	Yes
4-Methylvaleric acid	1.27	0.035	No
Methyl mercaptan	1.26	0.035	No
Linoleic acid	1.24	0.016	**
Propionamide	1.23	0.128	No
1-Dodecene	1.21	0.016	**
Butyric acid	1.15	0.289	Yes
3-Phenylpropionic acid	1.14	0.028	No
4-Vinylphenol	1.13	0.065	**
Acetic acid	1.07	0.065	Yes

* Based on the *t*-test between water with and without Salivette (*p* < 0.05). ** The compounds could not be confirmed as blanks because they were not detected in blank analysis (using water as a sample).

## Data Availability

Data available in a publicly accessible repository. The raw GC-MS data from this study have been deposited in MB-POST (https://repository.massbank.jp/preview/122243149068d5f26c3390a, accessed on 9 October 2025, PIN CODE: 9541).

## Supplementary Materials

The following supporting information can be downloaded at: https://www.mdpi.com/article/10.3390/metabo15110726/s1, Figure S1: Representative image of glandular saliva collection; Figure S2: Paired scatter plots of 10 compounds showing significant changes (*q* < 0.05, Wilcoxon signed-rank test) between glandular and whole saliva; Table S1: List of 554 compounds registered to the in-house library; Table S2: Compounds detected in the whole saliva treated with and without Salivette; Table S3: Compounds detected in glandular and whole saliva (excluding 15 compounds significantly changed upon using Salivette).

## Abbreviations

The following abbreviations are used in this manuscript:

VOCs	Volatile organic compounds
GC	Gas chromatography
MS	Mass spectrometry
RI	Retention index
OPLS-DA	Orthogonal partial least squares discriminant analysis
VSCs	Volatile sulfur compounds
VIP	Variable importance for prediction
