# Supplementary material for: Comprehensive GC/MS Profiling of Volatile Organic Compounds in Whole and Glandular Saliva Using MonoTrap Micro-Extraction
Source: Metabolites. 2025 Nov 6;15(11):726. doi: 10.3390/metabo15110726 (PMC12654116; doi:10.3390/metabo15110726)
Supplement: Supplementary file 1 [file metabolites-15-00726-s001.zip › metabolites-3946623-supplementary.pdf]

Supplementary materials

List of contents

Figure S1. Representative image of glandular saliva collection. 2

Figure S2. Paired scatter plots of 10 compounds showing significant changes (*q* < 0.05, Wilcoxon signed-rank test) between glandular and whole saliva. 3

Table S1. List of 554 compounds registered to the in-house library...4

Table S2. Compounds detected in the whole saliva treated with and without Salivette. 16

Table S3. Compounds detected in glandular and whole saliva (excluding 15 compounds significantly changed upon using Salivette). 18

Figure S1. Representative image of glandular saliva collection.

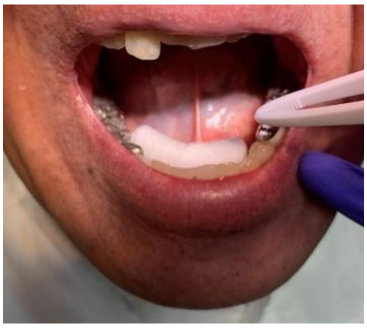

Figure S2. Paired scatter plots of 10 compounds showing significant changes ($q < 0.05$, Wilcoxon signed-rank test) between glandular and whole saliva.

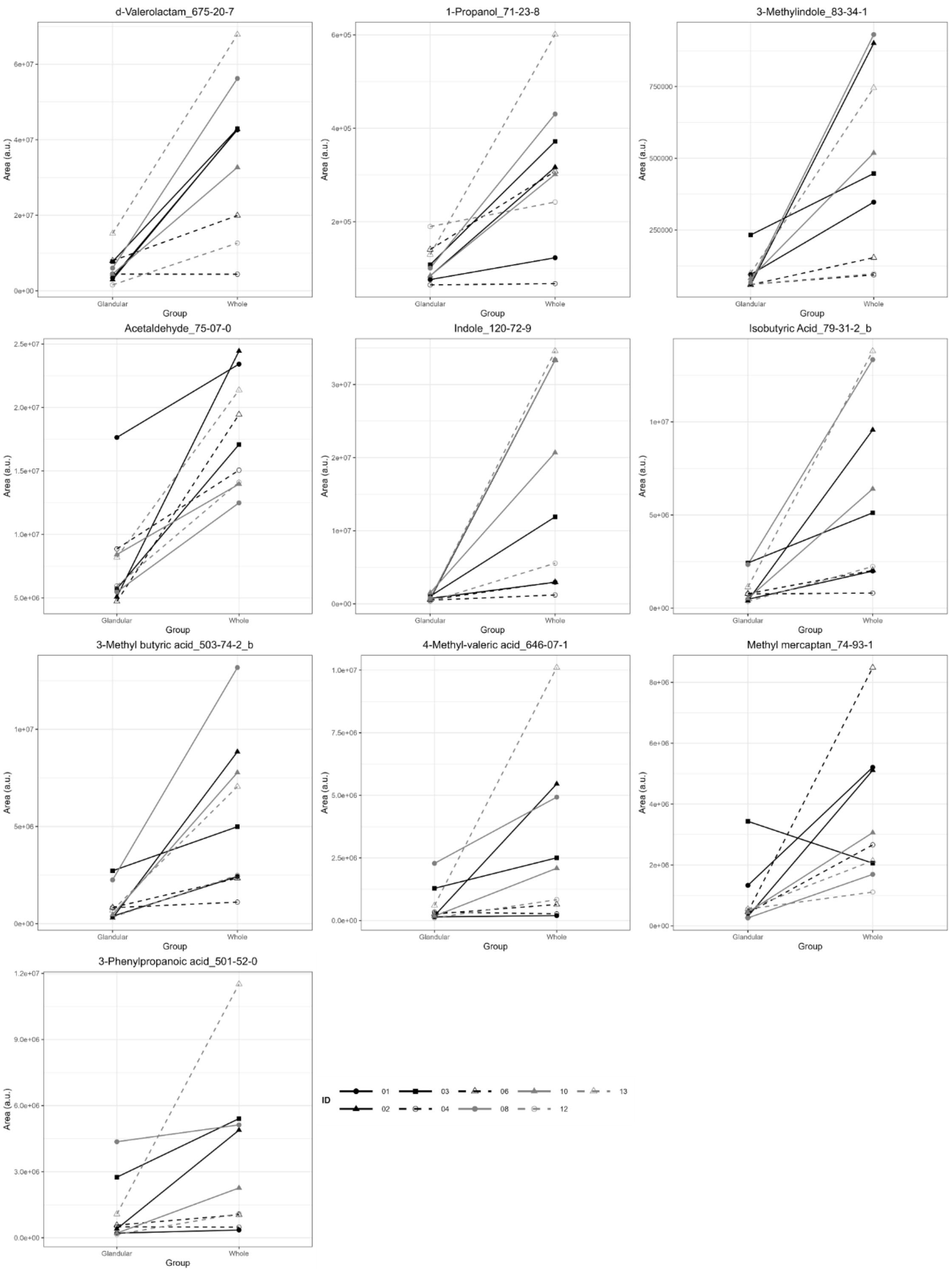

Table S1. List of 554 compounds registered to the in-house library.

No.	CAS registry number	Compound name
1	1002-84-2	Pentadecanoic acid
2	1003-29-8	Pyrrole-2-carboxaldehyde
3	100-41-4	Ethyl benzene
4	100-42-5	Styrene
5	100-47-0	Benzonitrile
6	100-51-6	Benzyl alcohol
7	100-52-7	Benzaldehyde
8	100-53-8	Benzyl mercaptan
9	101-41-7	Methyl phenylacetate
10	101-85-9	α -amylcinnamic alcohol
11	101-86-0	α -hexylcinnamaldehyde
12	101-97-3	Ethyl Phenylacetate
13	102-19-2	Phenylacetic acid isoamyl
14	103-26-4	Methyl cinnamate
15	103-36-6	Ethyl Cinnamate
16	103-41-3	Benzyl cinnamate
17	103-45-7	2-Phenethyl acetate
18	103-52-6	Phenethyl Butyrate
19	104-50-7	gamma-octalactone
20	104-54-1	cinnamyl alcohol
21	104-55-2	cinnamal
22	104-61-0	gamma-nonalactone
23	104-67-6	gamma-Undecalactone
24	104-76-7	2-Ethyl-1-hexanol
25	104-92-7	p-Bromoanisole
26	105-13-5	4-methoxybenzyl alcohol
27	105-37-3	Ethyl propanoate
28	105-46-4	sec-Butyl Acetate
29	105-54-4	Ethyl butyrate
30	105-60-2	Caprolactam
31	105-66-8	Propyl Butyrate
32	105-68-0	Isoamyl Propionate
33	105-79-3	Isobutyl Hexanoate
34	105-87-3	Geranyl acetate
35	106-22-9	Citronellol
36	106-24-1	Geraniol
37	106-25-2	Nerol
38	106-26-3	cis-Citral
39	106-27-4	Isoamyl Butyrate
40	106-30-9	Ethyl heptanoate
41	106-32-1	Ethyl Octanoate
42	106-33-2	Ethyl Laurate
43	106-33-2	Ethyl dodecanoate
44	106-35-4	3-Heptanone
45	106-36-5	Propyl Propionate
46	106-68-3	3-octanone
47	106-70-7	Methyl hexanoate

48	107-02-8	2-Propenal
49	107-18-6	Allyl Alcohol
50	1072-83-9	2-Acetylpyrrole
51	107-75-5	Hydroxycitronellal
52	107-87-9	2-Pentanone
53	107-88-0	1,3-Butanediol
54	107-92-6	Butyric acid
55	108-10-1	4-Methyl-2-pentanone
56	108-21-4	Isopropyl Acetate
57	108-29-2	gamma-Valerolactone
58	108-50-9	2,6-Dimethylpyrazine
59	108-59-8	Dimethyl malonate
60	108-64-5	Ethyl Isovalerate
61	108-65-6	1-Methoxy-2-propyl acetate
62	108-75-8	2,4,6-Collidine
63	108-88-3	Toluene
64	108-95-2	Phenol
65	109-08-0	2-Methylpyrazine
66	109-19-3	Butyl Isovalerate
67	109-21-7	Butyl Butyrate
68	109-52-4	Pentanoic acid
69	109-60-4	propyl acetate
70	109-89-7	Diethylamine
71	109-94-4	Ethyl formate
72	110-00-9	Furan
73	110-02-1	Thiophene
74	110-19-0	Isobutyl acetate
75	110-38-3	Ethyl Decanoate
76	110-39-4	Octyl butyrate
77	110-43-0	2-heptanone
78	110-62-3	Valeraldehyde
79	110-86-1	Pyridine
80	110-93-0	6-methyl-5-hepten-2-one
81	111-02-4	Squalene
82	111-11-5	Methyl n-Caprylate
83	111-12-6	methyl 2-octynoate
84	111-13-7	2-octanone
85	111-14-8	Heptanoic acid
86	111-27-3	1-Hexanol
87	111-35-3	3-Ethoxy-1-propanol
88	111-61-5	Ethyl octadecanoate
89	111-65-9	n-Octane
90	111-66-0	1-Octene
91	111-70-6	1-heptanol
92	111-71-7	Heptanal
93	1117-55-1	Hexyl octanoate
94	111-80-8	methyl 2-nonynoate
95	111-82-0	Methyl Laurate
96	111-84-2	n-Nonane
97	111-87-5	1-octanol

98	111-92-2	Dibutylamine
99	1120-21-4	n-Undecane
100	1120-36-1	1-Tetradecene
101	112-05-0	Pelargonic acid
102	112-06-1	Heptyl Acetate
103	112-12-9	2-Undecanone
104	112-14-1	Octyl acetate
105	112-30-1	1-Decanol
106	112-31-2	1-Decanal
107	112-37-8	Undecanoic Acid
108	112-40-3	n-Dodecane
109	1124-11-4	2,3,5,6-Tetramethylpyrazine
110	112-41-4	1-Dodecene
111	112-42-5	1-Undecanol
112	112-53-8	1-Dodecanol
113	112-63-0	Methyl Linoleate
114	112-80-1	Oleic Acid
115	112-88-9	1-Octadecene
116	112-95-8	n-Eicosane
117	1139-30-6	(-)-Caryophyllene oxide
118	115-18-4	2-methyl-3-buten-2-ol
119	115-95-7	Linalyl Acetate
120	116-09-6	Acetol
121	116-53-0	DL-2-Methylbutyric acid
122	118-58-1	benzyl salicylate
123	118-71-8	3-Hydroxy-2-methyl-4-pyrone
124	1192-62-7	2-Acetylfuran
125	1193-11-9	2,2,4-Trimethyl-1,3-dioxolane
126	119-36-8	Methyl salicylate
127	1193-79-9	2-Acetyl-5-methylfuran
128	1196-01-6	(-)-Verbenone
129	120-50-3	Isobutyl Benzoate
130	120-51-4	benzyl benzoate
131	120-72-9	Indole
132	1211-29-6	Methyl jasmonate
133	121-32-4	3-Ethoxy-4-hydroxybenzaldehyde
134	121-33-5	vanillin
135	122-00-9	4'-Methylacetophenone
136	122-40-7	alpha-Amylcinnamaldehyde
137	122-59-8	phenoxyacetic acid
138	122-70-3	2-Phenylethyl Propionate
139	122-78-1	Phenylacetaldehyde
140	123-07-9	4-Ethylphenol
141	123-08-0	4-Hydroxybenzaldehyde
142	123-11-5	p-Anisaldehyde
143	123-25-1	Diethyl succinate
144	123-29-5	Ethyl nonanoate
145	123-31-9	1,4-Benzenediol
146	123-32-0	2,5-dimethylpyrazine
147	123-38-6	Propionaldehyde

148	123-51-3	3-Methyl-1-butanol
149	123-54-6	Acetylacetone
150	123-66-0	Ethyl Hexanoate
151	123-72-8	Butyraldehyde
152	123-86-4	butyl acetate
153	123-92-2	3-methylbutyl acetate
154	123-96-6	(±)-2-Octanol
155	124-06-1	Ethyl tetradecanoate
156	124-07-2	Octanoic acid
157	124-10-7	Methyl Myristate
158	124-11-8	1-Nonene
159	124-13-0	1-Octanal
160	124-18-5	n-Decane
161	124-19-6	Nonanal
162	124-76-5	Isoborneol
163	125-12-2	Isobornyl acetate
164	126-91-0	(R)-Linalool
165	127-41-3	alpha-Ionone
166	127-51-5	iso-α-methylionone
167	128-37-0	2,6-Di-tert-butyl-p-cresol (butylated hydroxytoluene)
168	13327-56-5	Ethyl 3-(Methylthio)propionate
169	13360-65-1	2-Ethyl-3.6-dimethylpyrazine
170	13419-69-7	trans-2-Hexenoic Acid
171	134-20-3	Methyl 2-Aminobenzoate
172	134-96-3	Syringaldehyde
173	13679-70-4	5-Methylthiophene-2-carboxaldehyde
174	136954-20-6	3-Sulfanylhexyl acetate
175	137-32-6	DL-2-Methyl-1-butanol
176	13925-00-3	2-ethyl-pyrazine
177	13925-07-0	2-Ethyl-3.5-dimethylpyrazine
178	140-10-3	trans-Cinnamic acid
179	140-11-4	Benzyl acetate
180	140-26-1	2-Phenylethyl Isovalerate
181	14059-92-8	4-Ethyl-2,6-dimethoxyphenol
182	140-67-0	4-allylanisole
183	14073-97-3	L-menthone
184	141-12-8	Neryl Acetate
185	141-27-5	trans-Citral
186	141-78-6	Ethyl Acetate
187	141-79-7	4-Methyl-3-penten-2-one
188	14250-96-5	(E)-2-Ethylpent-2-enal
189	142-62-1	hexanoic acid
190	142-82-5	n-Heptane
191	142-92-7	Hexyl Acetate
192	143-07-7	Lauric Acid
193	143-08-8	1-Nonanol
194	14436-32-9	9-Decenoic acid
195	14667-55-1	2,3,5-Trimethylpyrazine
196	14901-07-6	β-Ionone
197	14936-66-4	2-Nonyl Acetate

198	149-57-5	2-ethylhexanoic acid
199	150-13-0	4-aminobenzoic acid
200	150-86-7	Phytol
201	1534-08-03	S-Methyl Thioacetate
202	1565-80-6	(S)-(-)-2-Methyl-1-butanol
203	15706-73-7	Butyl 2-Methylbutyrate
204	15707-23-0	2-Ethyl-3-methylpyrazine
205	1576-87-0	trans-2-Pentenal
206	1576-95-0	cis-2-Penten-1-ol
207	1577-18-0	trans-3-Hexenoic acid
208	1629-58-9	1-Penten-3-one
209	1632-73-1	Fenchyl Alcohol
210	1653-30-1	2-Undecanol
211	16630-52-7	3-(methylthio)butyraldehyde
212	1669-44-9	3-Octen-2-one
213	1679-47-6	alpha-Methyl-g-butylrolactone
214	1731-84-6	Methyl Nonanoate
215	1741-41-9	Isobutyraldehyde Diethyl Acetal
216	18172-67-3	(-)-beta-Pinene
217	18281-05-5	Ethyl eicosanoate
218	18309-28-9	D-isomenthone
219	18409-17-1	trans-2-Octen-1-ol
220	18794-84-8	trans-beta-Farnesene
221	18829-55-5	trans-2-Heptenal
222	18829-56-6	trans-2-Nonenal
223	19322-27-1	4-Hydroxy-5-methyl-3-furanone (Norfuraneol)
224	19329-89-6	Isoamyl Lactate
225	20125-84-2	cis-3-Octen-1-ol
226	20126-76-5	(-)-Terpinen-4-ol
227	2021-28-5	Ethyl 3-phenylpropionate
228	2035-99-6	Isoamyl n-Octanoate
229	2050-01-3	Isoamyl Isobutyrate
230	2050-09-1	Isoamyl Valerate
231	20582-85-8	4-(Methylthio)butanol
232	2179-60-4	Methyl Propyl Disulfide
233	21834-92-4	5-Methyl-2-phenyl-2-hexenal
234	2198-61-0	Isoamyl Hexanoate
235	22047-25-2	2-Acetylpyrazine
236	22122-36-7	3-methyl-2(5H)-furanone
237	2217-02-9	(1R)-endo-(+)-Fenchyl alcohol
238	2244-16-8	(S)-(+)-Carvone
239	2305-05-7	gamma-Dodecanolactone
240	2306-91-4	Isoamyl Decanoate
241	23089-26-1	(-)-alpha-Bisabolol
242	2311-46-8	Isopropyl hexanoate
243	2345-28-0	2-Pentadecanone
244	23696-85-7	Damascenone
245	23726-91-2	(E)-β-Damascone
246	24295-03-2	2-Acetylthiazole
247	2432-51-1	S-Methyl Thiobutyrate

248	2437-56-1	1-Tridecene
249	2445-77-4	2-Methylbutyl isovalerate
250	24634-95-5	Ethyl tetracosanoate
251	2497-18-9	trans-2-Hexenyl Acetate
252	25152-84-5	2,4-Decadienal
253	25415-67-2	Ethyl 4-methylpentanoate
254	25415-84-3	2-Ethylhexyl Butyrate
255	2548-87-0	trans-2-Octenal
256	25773-40-4	2-Isopropyl-3-methoxypyrazine
257	2628-17-3	4-Vinylphenol
258	2639-63-6	Hexyl Butyrate
259	26549-24-6	(R)-(-)-2-Hexanol
260	27538-09-6	Homofuraneol
261	27625-35-0	3-Methylbutyl 2-methylbutanoate
262	2785-89-9	2-Methoxy-4-ethylphenol
263	28588-74-1	2-Methyl-3-furanthiol
264	28588-75-2	bis(2-methyl-3-furanyl) disulfide
265	28664-35-9	3-hydroxy-4,5-dimethyl-2(5H)-furanone
266	288-13-1	Pyrazole
267	288-47-1	Thiazole
268	290-37-9	Pyrazine
269	29548-30-9	Farnesyl Acetate
270	2983-38-2	Ehtyl 2-ethyl butanoate
271	301-00-8	Methyl Linolenate
272	3050-69-9	Vinyl n-Hexanoate
273	31906-04-4	Lyral
274	3208-16-0	2-Ethylfuran
275	321-38-0	1-fluoronaphthalene
276	3221-61-2	2-methyloctane
277	3268-49-3	Methional
278	3338-55-4	cis-B-Ocimene
279	334-48-5	Decanoic Acid
280	3387-41-5	Sabinene
281	3391-86-4	1-octen-3-ol
282	34300-94-2	3-Sulfanyl-3-methyl-1-butanol
283	34995-77-2	trans-Linalool oxide
284	35154-45-1	cis-3-Hexenyl Isovalerate
285	35192-73-5	1-Nonen-4-ol
286	35897-13-3	3-Methylpentyl acetate
287	3658-77-3	2,5-Dimethyl-4-hydroxy-3(2H)-furanone
288	3658-80-8	Dimethyl Trisulfide
289	3681-71-8	cis-3-Hexenyl Acetate
290	3777-69-3	2-Pentylfuran
291	3779-61-1	trans-B-Ocimene
292	3790-78-1	cis-Nerolidol
293	3848-24-6	2,3-Hexanedione
294	39638-67-0	trans-Whiskey lactone
295	40716-66-3	trans-Nerolidol
296	4077-47-8	2,5-Dimethyl-4-methoxy-3(2H)-furanone
297	40789-98-8	3-Mercapto-2-butanone

298	4170-30-3	2-Buten-1-al
299	4170-30-3	Crotonaldehyde
300	431-03-8	2,3-butanedione
301	4312-99-6	1-Octen-3-one
302	4313-03-5	(E,E)-2,4-Heptadienal
303	4407-36-7	Cinnamyl alcohol
304	4411-89-6	2-Phenyl-2-butenal
305	4435-53-4	3-Methoxybutyl Acetate
306	4437-51-8	3,4-Hexanedione
307	4466-24-4	2-Butylfuran
308	4602-84-0	Farnesol
309	4630-07-3	Valencene
310	463-40-1	Linolenic Acid
311	464-43-7	(+)-Borneol
312	464-45-9	(-)-Borneol
313	4674-50-4	(+)-Nootkatone
314	469-61-4	(-)-a-Cedrene
315	470-82-6	1,8-Cineole
316	489-86-1	(-)-Guaiol
317	4906-24-5	3-Acetoxy-2-butanone
318	4938-52-7	1-Hepten-3-ol
319	4940-11-8	2-Ethyl-3-hydroxy-4-pyrone
320	497-03-0	trans-2-Methyl-2-butenal
321	497-23-4	2(5H)-furanone
322	498-15-7	3-Carene
323	499-75-2	Carvacrol
324	501-52-0	3-Phenylpropanoic acid
325	501-94-0	2-(4-Hydroxyphenyl)ethyl Alcohol
326	502-44-3	epsilon-Caprolactone
327	503-74-2	3-Methyl butyric acid
328	504-63-2	1,3-Propanediol
329	505-10-2	Methionol
330	505-32-8	Isophytol
331	506-12-7	Heptadecanoic acid
332	5077-67-8	1-hydroxy-2-butanone
333	513-85-9	2,3-Butanediol
334	513-86-0	Acetoin
335	51755-83-0	3-Mercapto-1-hexanol
336	5271-38-5	2-(Methylthio)ethanol
337	527-84-4	o-Isopropyltoluene
338	53398-83-7	trans-2-Hexenyl butyrate
339	53398-85-9	cis-3-Hexen-1-yl 2-Methylbutyrate
340	534-22-5	2-Methylfuran
341	53448-07-0	trans-2-Undecenal
342	535-77-3	m-Isopropyltoluene
343	538-68-1	Amylbenzene
344	5392-40-5	citral
345	539-82-2	Ethyl n-Valerate
346	539-90-2	Isobutyl Butyrate
347	540-18-1	Amyl Butyrate

348	541-47-9	3-Methylcrotonic acid
349	543-49-7	2-Heptanol
350	544-01-4	Isoamyl Ether
351	544-63-8	Myristic Acid
352	544-85-4	n-Dotriacontane
353	546-79-2	Sabinene Hydrate
354	546-80-5	(-)-alpha-Thujone
355	5471-51-2	Raspberry ketone
356	547-63-7	Methyl Isobutyrate
357	55013-32-6	cis-Whiskey lactone
358	551-08-6	3-Butylidenephthalide
359	551-93-9	2'-Aminoacetophenone
360	554-12-1	Methyl Propionate
361	556-24-1	Methyl 3-methylbutanoate
362	556-82-1	Prenol
363	557-48-2	(E,Z)-2,6-Nonadienal
364	562-74-3	Terpinen-4-ol
365	563-80-4	3-Methyl-2-butanone
366	565-63-9	(Z)-2-methylbut-2-enoic acid
367	57-10-3	Palmitic Acid
368	57-11-4	Stearic acid
369	58-08-2	Caffeine
370	58175-57-8	2-Propyl-1-pentanol
371	582-24-1	2-Hydroxyacetophenone
372	5834-16-2	3-Methylthiophene-2-carboxaldehyde
373	586-62-9	Terpinolene
374	589-38-8	3-Hexanone
375	589-59-3	Isobutyl Isovalerate
376	589-75-3	Butyl n-Octanoate
377	589-82-2	3-Heptanol
378	589-98-0	3-Octanol
379	590-01-2	Butyl Propionate
380	590-36-3	2-Pentanol, 2-methyl-
381	590-86-3	3-methylbutanal
382	5908-87-2	Ethyl docosanoate
383	5910-87-2	trans, trans-2,4-Nonadienal
384	5910-89-4	2,3-dimethylpyrazine
385	591-68-4	Butyl Valerate
386	591-78-6	2-Hexanone
387	593-08-8	2-Tridecanone
388	593-45-3	n-Octadecane
389	593-49-7	n-Heptacosane
390	59-67-6	Nicotinic Acid
391	5989-27-5	(R)-(+)-Limonene
392	599-04-2	Pantolactone
393	600-14-6	2,3-Pentanedione
394	60-12-8	2-phenylethanol
395	60-24-2	Mercaptoethanol; 2-Mercaptoethanol
396	6032-29-7	2-Pentanol
397	60-33-3	Linoleic acid

398	614-75-5	2-Hydroxyphenylacetic acid
399	616-25-1	1-Penten-3-ol
400	620-02-0	5-Methyl-2-furaldehyde
401	623-17-6	Furfuryl acetate
402	623-19-8	Furfuryl propionate
403	623-37-0	3-Hexanol
404	623-42-7	Methyl butyrate
405	624-24-8	Methyl valerate
406	624-41-9	2-Methylbutyl acetate
407	624-89-5	Ethyl Methyl Sulfide
408	624-92-0	Dimethyl Disulfide
409	625-33-2	3-Penten-2-one
410	625-60-5	S-Ethyl Thioacetate
411	625-86-5	2,5-Dimethylfuran
412	626-38-0	1-Methylbutyl acetate
413	626-82-4	Butyl Hexanoate
414	628-63-7	Amyl Acetate
415	628-73-9	Hexanenitrile
416	628-97-7	Hexadecanoic acid, ethyl ester
417	628-99-9	2-Nonanol
418	629-19-6	Dipropyl Disulfide
419	629-50-5	n-Tridecane
420	629-59-4	n-Tetradecane
421	629-62-9	Pentadecane
422	629-76-5	1-Pentadecanol
423	629-78-7	n-Hexadecane
424	629-92-5	n-Nonadecane
425	629-94-7	n-Heneicosane
426	629-97-0	n-Docosane
427	629-99-2	n-Pentacosane
428	630-01-3	n-Hexacosane
429	630-02-4	n-Octacosane
430	630-03-5	n-Nonacosane
431	630-04-6	n-Hentriacontane
432	6378-65-0	Hexyl Hexanoate
433	637-92-3	tert-Butyl Ethyl Ether
434	638-11-9	Isopropyl butyrate
435	638-36-8	Phytane (2,6,10,14-Tetramethylhexadecane)
436	638-67-5	n-Tricosane
437	638-68-6	n-Triacontane
438	64-17-5	Ethanol
439	64-18-6	Formic acid
440	64-19-7	Acetic acid
441	646-01-5	3-Methylthiopropionic Acid
442	646-07-1	4-Methyl-valeric acid
443	646-31-1	n-tetracosane
444	65505-17-1	2-Methyl-3-(methyldithio)furan
445	65-85-0	benzoic acid
446	659-70-1	Isoamyl Isovalerate
447	66-25-1	Hexanal

448	6728-26-3	trans-2-Hexenal
449	674-26-0	4-Hydroxy-4-methyltetrahydro-2H-pyran-2-one
450	67-47-0	5-(Hydroxymethyl)-2-furfural
451	675-20-7	d-Valerolactam
452	6753-98-6	alpha-Humulene
453	67-56-1	Methanol
454	67-63-0	2-propanol
455	67-64-1	Acetone
456	67-66-3	Chloroform
457	687-47-8	(-)-Ethyl L-lactate
458	695-06-7	5-Ethyldihydro-2(3H)-Furanone
459	698-10-2	Maple furanone
460	698-76-0	delta-Octalactone
461	706-14-9	gamma-Decalactone
462	71-23-8	1-Propanol
463	71-36-3	1-Butanol
464	71-41-0	1-Pentanol
465	7212-44-4	Nerolidol
466	7367-88-6	Ethyl trans-2-decenoate
467	7452-79-1	Ethyl DL-2-Methylbutyrate
468	74-93-1	Methyl Mercaptan Standard Solution
469	75-05-8	Acetonitrile
470	75-07-0	Acetaldehyde
471	75-08-1	Ethyl Mercaptan
472	75-09-2	dichloromethane
473	75-18-3	Dimethyl Sulfide
474	75-65-0	tert-Butyl Alcohol
475	75-98-9	Pivalic Acid
476	76-22-2	Camphor
477	763-29-1	1-Pentene, 2-methyl-
478	763-32-6	3-Methyl-3-buten-1-ol
479	765-70-8	3-Methyl-1,2-cyclopentanedione
480	76649-16-6	Ethyl trans-4-Decenoate
481	77-53-2	Cedrol
482	7786-61-0	2-Methoxy-4-vinylphenol
483	7787-20-4	(1R)-(-)-Fenchone
484	78-70-6	Linalool
485	78-79-5	Isoprene
486	78-83-1	2-Methyl-1-propanol
487	78-84-2	Isobutyraldehyde
488	78-92-2	2-Butanol
489	78-93-3	2-Butanone
490	79-05-0	Propionamide
491	79-09-4	Propionic Acid
492	79-10-7	Acrylic acid
493	79-20-9	Methyl acetate
494	79-31-2	Isobutyric Acid
495	79-50-5	DL-Pantolactone
496	79-92-5	Camphene
497	80-54-6	lilial

498	80-56-8	alpha-Pinene
499	80-59-1	(E)-2-Methyl-2-butenoic Acid
500	80-71-7	2-Hydroxy-3-methyl-2-cyclopentenone
501	819-97-6	sec-Butyl Butyrate
502	821-55-6	2-Nonanone
503	822-36-6	4-Methylimidazole
504	83-34-1	3-Methylindole
505	868-57-5	Methyl 2-methylbutanoate
506	872-05-9	1-Decene
507	87-44-5	beta-Caryophyllene
508	88-09-5	2-Ethylbutyric acid
509	88-15-3	2-Acetylthiophene
510	89-78-1	Menthol
511	89-79-2	(-)-Isopulegol
512	89-81-6	Piperitone
513	89-83-8	Thymol
514	90-05-1	2-Methoxyphenol(Guaiacol)
515	91-20-3	Naphthalene
516	91-64-5	coumarin
517	925-78-0	3-Nonanone
518	928-80-3	3-Decanone
519	928-91-6	cis-4-Hexen-1-ol
520	928-92-7	4-Hexen-1-ol,predominantly trans
521	928-95-0	trans-2-Hexen-1-ol
522	928-96-1	cis-3-Hexen-1-ol
523	928-97-2	trans-Hexen-1-ol
524	93-15-2	methyl eugenol
525	93-51-6	2-Methoxy-4-methylphenol
526	93-53-8	2-Phenylpropionaldehyde
527	93-58-3	Methyl Benzoate
528	93-89-0	ethyl benzoate
529	94-46-2	Isoamyl benzoate
530	94-59-7	safrole
531	95-16-9	Benzothiazole
532	96-04-8	2,3-Heptanedione
533	96-17-3	2-Methylbutanal
534	96-22-0	3-Pentanone
535	96-29-7	2-Butanone Oxime
536	96-48-0	Gamma-Butyrolactone
537	97-53-0	eugenol
538	97-54-1	isoeugenol
539	97-62-1	Ethyl Isobutyrate
540	97-64-3	Ethyl Lactate
541	97-85-8	Isobutyl Isobutyrate
542	97-87-0	Butyl Isobutyrate
543	98-00-0	Furfuryl alcohol，2-Furanylmethanol
544	98-01-1	Furfural
545	98-02-2	Furfuryl Mercaptan
546	98-03-3	2-Thiophenecarboxaldehyde
547	98-55-5	a-Terpineol

548	98-85-1	1-Phenylethyl alcohol
549	98-86-2	Acetophenone
550	99-76-3	Methyl4-hydroxybenzonate
551	99-83-2	alpha-Phellandrene
552	99-85-4	gamma-Terpinene
553	99-86-5	alpha-Terpinene
554	99-87-6	p-Cymene

Table S2. Compounds detected in the whole saliva treated with and without Salivette.

No.	RT* (min)	RI	Quant mass	Compound	Average intensity**	RSD (%)***
				Alcohol		
1	7.6	933	45	2-Propanol	334540	15.6
2	11.4	1051	31	1-Propanol	87407	26.2
3	16.2	1167	56	1-Butanol	49319	11.8
4	18.9	1226	57	2-Methyl-1-butanol	77480	25.0
5	19	1227	55	3-Methyl-1-butanol	49350	23.8
6	19.5	1239	69	2-Hexanol	2926	12.5
7	21	1268	55	1-Pentanol	14401	18.6
8	23.8	1328	43	Acetol	177926	18.8
9	25.6	1367	56	1-Hexanol	19286	9.3
10	31.6	1499	57	2-Ethyl-1-hexanol	1790918	16.5
11	38	1653	81	Menthol	8312	16.7
12	47.1	1896	79	Benzyl alcohol	17301	1.2
13	48.4	1932	91	2-Phenylethanol	619805	38.7
				Aromatic compound		
14	4.8	806	68	Furan	31406	3.4
15	6.1	878	82	2-Methylfuran	14792	11.1
16	18.3	1213	79	Pyridine	459033	14.7
17	22.2	1294	94	2-Methylpyrazine	11653	13.1
18	31.2	1490	96	Furfural	63586	8.2
19	32.9	1530	95	2-Acetylfuran	10813	7.8
20	33.8	1552	106	Benzaldehyde	46120	29.1
21	35.9	1600	110	5-Methyl-2-furaldehyde	69521	10.0
22	38.9	1677	98	Furfuryl alcohol	139930	8.8
23	58.7	2251	82	4-Methylimidazole	59792	18.9
24	65.2	2473	105	Benzoic acid	98367	6.1
25	65.3	2477	117	Indole	3728041	6.4
26	66.6	2524	130	Skatole	80368	1.2
27	66.8	2530	97	5-Hydroxymethyl-2-furaldehyde	13567	11.9
28	68.7	2601	151	Vanillin	7975	14.7
29	70	2636	91	3-Phenylpropionic acid	1257593	10.5
30	89.4	3206	194	Caffeine	8267813	1.3
				Aldehyde		
31	3.8	709	44	Acetaldehyde	6596422	8.2
32	4.8	804	58	Propionaldehyde	50233	11.8
33	5	819	43	Isobutyraldehyde	123352	6.3
34	5.8	858	56	2-Propenal	12636	6.7
35	7.2	922	57	2-Methylbutyraldehyde	53855	20.6
36	7.3	926	44	3-Methylbutanal	135964	8.7
				Fatty acid		
37	30.3	1469	43	Acetic acid	8397243	0.02
38	33.9	1554	74	Propionic acid	5814918	18.0
39	35.1	1581	43	Isobutyric acid	1364091	20.8
40	37.6	1643	60	Butyric acid	3265335	20.4
41	39.1	1682	60	Isovaleric acid	1743843	21.0

42	41.8	1752	60	Valeric acid	81619	16.2
43	44.2	1816	57	4-Methylvaleric acid	623534	19.2
44	45.7	1858	60	Hexanoic acid	90836	19.5
45	49.5	1964	60	Heptanoic acid	36354	17.2
46	53.1	2071	60	Octanoic acid	195953	17.9
47	56.4	2177	60	Pelargonic acid	683691	11.7
48	59.7	2283	73	Decanoic acid	126361	10.6
49	65.8	2494	73	Lauric acid	154966	9.3
				Ketone		
50	5.1	825	43	Acetone	584242	6.4
51	6.9	912	43	2-Butanone	98264	8.9
52	9.3	993	43	2,3-Butanedione	123784	13.8
53	10.3	1020	43	4-Methyl-2-pentanone	30158	10.8
54	16.5	1174	57	3-Heptanone	17688	8.4
55	23	1310	45	Acetoin	200651	18.6
56	39	1679	105	Acetophenone	33549	17.1
				VSC		
57	3.7	698	47	Methyl mercaptan	622411	13.1
58	27.3	1402	126	Dimethyl trisulfide	15122	13.7
59	50.3	1990	135	Benzothiazole	12052	25.2
				Others		
60	6.5	899	43	Ethyl acetate	172955	34.1
61	44.7	1829	44	Propionamide	75082	1.6
62	56.1	2167	30	δ-Valerolactam	8039670	2.7

* Retention time.

** These data are based on $n = 3$ (technical replicates) of whole saliva without Salivette treatment.

*** Relative standard deviation. These data are based on $n = 3$ (technical replicates) of whole saliva without Salivette treatment.

Table S3. Compounds detected in glandular and whole saliva (excluding 15 compounds significantly changed upon using Salivette).

No.	RT (min)	RI	Quant mass	Compound
			Alcohol	
1	7.6	933	45	2-Propanol
2	11.5	1050	31	1-Propanol
3	16.3	1167	56	1-Butanol
4	19	1226	57	2-Methyl-1-butanol
5	19	1226	55	3-Methyl-1-butanol
6	21	1267	55	1-Pentanol
7	23.9	1327	43	Acetol
8	31.7	1498	57	2-Ethyl-1-hexanol
9	38.1	1653	71	Menthol
10	47.2	1896	79	Benzyl alcohol
11	48.5	1932	91	2-Phenylethanol
			Aromatic compound	
12	6.2	878	82	2-Methylfuran
13	18.3	1211	79	Pyridine
14	19.7	1240	80	Pyrazine
15	20.1	1247	81	2-Pentylfuran
16	22.2	1292	94	2-Methylpyrazine
17	28.5	1426	122	2,3,5-Trimethylpyrazine
18	33.9	1551	106	Benzaldehyde
19	35.9	1599	110	5-Methyl-2-furaldehyde
20	58.7	2246	82	4-Methylimidazole
21	63.7	2416	120	4-Vinylphenol
22	65.4	2474	105	Benzoic acid
23	65.5	2477	117	Indole
24	66.8	2524	130	Skatole
25	70.1	2635	91	3-Phenylpropionic acid
26	89.6	3203	194	Caffeine
			Aldehyde	
27	3.9	709	44	Acetaldehyde
28	4.8	803	58	Propionaldehyde
29	5.1	819	43	Isobutyraldehyde
30	5.8	857	56	2-Propenal
31	7.3	922	57	2-Methylbutyraldehyde
32	7.4	926	44	3-Methylbutanal
			Fatty acid	
33	30.4	1468	43	Acetic acid
34	34	1554	28	Propionic acid
35	34.4	1561	46	Formic acid
36	35.2	1581	43	Isobutyric acid
37	37.7	1642	60	Butyric acid
38	39.3	1682	60	Isovaleric acid
39	44.3	1816	57	4-Methylvaleric acid
40	53.2	2071	60	Octanoic acid
41	65.9	2493	73	Lauric acid
42	90.8	3239	67	Linoleic acid

		Ketone		
43	5.2	824	43	Acetone
44	7	912	43	2-Butanone
45	9.4	992	43	2,3-Butanedione
46	10.3	1019	43	4-Methyl-2-pentanone
47	16.6	1173	57	3-Heptanone
48	23	1309	45	Acetoin
		VSC		
49	3.7	698	47	Methyl mercaptan
50	13.1	1094	94	Dimethyl disulfide
51	27.4	1402	126	Dimethyl trisulfide
52	29.8	1455	81	Furfuryl mercaptan
		Others		
53	6.6	899	43	Ethyl acetate
54	20	1247	55	1-Dodecene
55	44.7	1827	44	Propionamide
56	56.2	2164	99	δ-Valerolactam
57	57.2	2197	135	Thymol
